# In-Law and Mate Preferences in Chinese Society and the Role of Traditional Cultural Values

**DOI:** 10.1177/1474704917730518

**Published:** 2017-09-13

**Authors:** Qingke Guo, Yujie Li, Shushuang Yu

**Affiliations:** 1 Department of Psychology, Shandong Normal University, Jinan, People’s Republic of China

**Keywords:** parent–child divergence, mate preference, evolutionary psychology, cultural universal, cultural specific

## Abstract

Using 347 parent–child dyads as participants, this study directly examined in-law and mate preferences in a typical collectivist culture. The results showed (1) traits indicating social status and parental investment were more highly valued by the parents, while traits indicating genetic quality and traits related to romantic love were more highly valued by the children. (2) Parental preferences were moderated by gender of the in-laws. Good earning capacity was more preferred by parents in a son-in-law, traits connoting genetic quality and reproductive fitness were more preferred by parents in a daughter-in-law. (3) There was more convergence in in-law and mate preferences in Chinese culture than in Western cultures. (4) Traditional cultural values (i.e., filial piety) can be used as a predictor of traditional mate preferences and less parent–child divergences. Additionally, greater preference for kind and understanding by parents than by children as well as by daughters than by sons, and greater preference for social status by the daughters’ than by the sons’ parents have not been observed in the rating and the ranking instrument. These findings illustrated how culture handles the parent–child disagreement over mating by authorizing greater parental influence on children’s mating decisions.

In most societies and historical periods, parents often have a strong influence on their children’s mating decisions because of their genetic relatedness to and power over their children (Perilloux, Fleischman, & Buss, 2008; Schlomer, Del Giudice, & Ellis, 2011). The selection of an in-law can bring tremendous survival and reproduction advantages to parents and their family. To maximize their genetic interests, parents prefer specific traits in a prospective in-law. However, the traits desired by the parents do not necessarily maximize the genetic interests of their children, given that parents and children have a coefficient of genetic relatedness of 0.50. Specifically, the parents prefer their child having a mate of high parental investment that is beneficial for all the parents’ children and grandchildren, and the child prefers having a mate of high genetic quality, because he or she may obtain more genetic benefits for his or her own offspring than the parents (Apostolou, 2016; Buunk, Park, & Dubbs, 2008; Trivers, 1974). This translates into agreement but also disagreement regarding what is considered an ideal mate (Schlomer et al., 2011; Trivers, 1974).

Parent–offspring divergence in mate preferences seems to be universal, but the degree to which children adopt parental preferences may also vary across cultures (Bejanyan, Marshall, & Ferenczi, 2014, 2015; Buunk, Park, & Duncan, 2010; Buunk, Pollet, & Dubbs, 2012; Dubbs, Buunk, & Taniguchi, 2013). As a crucial cultural dimension for understanding human behavior in different populations, individualism–collectivism (Hofstede, 1980) has been found to be strongly associated with the level of parental influence on mating (Bejanyan et al., 2014, 2015; Buunk et al., 2010; Schwartz et al., 2010). Individualistic cultures encourage independence and uniqueness, advocating self-determination, and personal choice. In individualistic cultures, the qualities seen as desirable in a mate are a personal matter. But in collectivist cultures, people tend to consider the well-being of the in-group over their own and are more likely to abandon personal desires that conflict with the in-group welfare (Buunk et al., 2008; Dubbs et al., 2013). Therefore, a high degree of parental influence on children’s mate choice seems more acceptable in collectivist cultures. China is a typical collectivist society characterized by high endorsement of Confucian filial piety (Ho, 1994; Nichol, 2013; Schwartz et al., 2010), a value system that originated from ancient China and is still influential in many Asian countries. By moralizing children’s obedience to family elders and authorizing parental control over the children’s mate preferences and mating strategies, filial piety may successfully solve an evolutionary problem for parents (Nichol, 2013). Children are socialized to be deferential and obedient to family elders, prioritizing the interests of family members and extended family members over their own. Children who violate filial obligations always experience negative emotions such as guilt and shame (Schwartz et al., 2010). This can lead to significantly greater harmony between parents and children compared with their Western counterparts. In this study, we used Chinese parent–child dyads as participants to directly compare their in-law and mate preferences. This will make a valuable addition to the emerging evolutionary psychological literature testing parent–offspring conflict theory (Schlomer et al., 2011; Trivers, 1974) in different cultural settings.

## Divergence Between In-Law and Mate Preferences

Parents and children are genetically related but not genetically identical. This translates into overlapping as well as diverging in-law and mate preferences. A child’s marriage can increase his or her parent’s inclusive fitness by (1) leading to the production of genetically high-quality grandchildren and (2) forging political alliances that increase the fitness of kin other than the betrothed. Because grandchildren are half as genetically related to grandparents as they are to parents, prospective grandparents will tend to weight the production of healthy grandchildren as relatively less important as prospective parents will (Apostolou, 2007a; Perilloux et al., 2008; Trivers, 1974). Genetic quality, as indexed by traits such as physical attractiveness, athleticism, creativity, and intelligence, can bring about asymmetric costs and benefits for parents and for children. Higher genetic quality in a mate may result in healthier, more attractive, and probably more successful offspring. But an individual can reap more genetic benefits from a genetically high-quality mate than his or her parents from a genetically high-quality in-law, because he or she share more genetic overlap with his or her own children (50%) than his or her parents with their grandchildren (25%). If children’s mate selection is based on their own preferences, they should give more weight to genetic quality in a potential mate than parents in an in-law (Apostolou, 2010a, 2015a; Buunk et al., 2008; Perilloux, Fleischman, & Buss, 2011; Schlomer et al., 2011). There are also characteristics (e.g., good family background) that are more preferred in an in-law than in a spouse (Buunk et al., 2008; Perilloux et al., 2011), because these characteristics can extend parents’ cooperative alliances and enhance their status, giving more fitness benefits to parents than to children. However, the discrepancy mentioned above may only make sense if there is a cost to the parents. That is, if a genetically high-quality mate is a bad parent, or if parents prioritize other traits over genetic quality.

The divergence in mate preferences between parents and children, especially between parents’ and daughters’, can also be understood in terms of evolutionary trade-offs (Gangestad & Simpson, 2000; Trivers, 1974). That is, pursuing one type of benefit inevitably reduces the likelihood of obtaining another type of benefit. From a biological point of view, the benefits provided by a mate can be grouped into two main clusters: heritable fitness and parental investment. High genetic quality in an in-law contributes to parents’ fitness through the birth of genetically high-quality offspring (i.e., the parents’ grandchildren). However, individuals of high genetic quality may probably be poorer quality parents, as they tend to invest more effort into seeking new mates than into raising children (Apostolou, 2016; Trivers, 1974). Mating with a partner of high genetic quality, but low investment potential may lead a daughter to depend on her parents and other kin for support. This will limit the parents’ ability to invest in their children and grandchildren. Moreover, an in-law of good investment potential can bring fitness interests to the parents and their family through protection and resources provision or childcare. As a consequence, when evaluating the suitability of a potential in-law, parents would place more value on traits indicating parental investment and cooperation with in-group compared with the children themselves.

Findings suggest that parents are more interested in controlling their daughters’ than their sons’ mating decisions (Apostolou, 2007b, 2010b, 2015b, 2017; Dubbs, Buunk, & Li, 2012; Dubbs et al., 2013; Perilloux et al., 2008). Female invests more in her offspring, so the trade-off between good-provider and good-genes selection is especially relevant for daughters (Buunk et al., 2008). So a son-in-law of good investment potential is more highly valued by the daughter’s parents than by the daughter herself. Selecting an investing son-in-law is especially important for survival of the daughters’ children, as parents are certain that they are genetically related to the offspring of their daughter than to that of their son. If a son-in-law is a poor investor, the parents are pressured to take the responsibility to invest in their daughter’s offspring themselves (Perilloux et al., 2008). Moreover, females are especially valuable reproductive resource in the mating markets to which males strive to access. While a sexual adventure can damage a daughter’s reputation and decrease her long-term mate value, leading to detrimental consequences for her parents’ fitness interests (Apostolou, 2009, 2015b; Perilloux et al., 2008). Therefore, a successful control of their daughters’ mating behaviors may enable the parents to maximize their own inclusive fitness by extracting more resources from their sons-in-law or by building new alliances and elevating social status. In some cultures, parents may even pressure their daughters to marry into a family with economic success and strong social standing (Apostolou, 2010a, b; Bejanyan et al., 2014, 2015). Therefore, we can infer that parents may be choosier in the selection of a son-in-laws versus a daughter-in-laws, as parents are more likely to exert stricter control over the daughters’ versus the sons’ mating decisions (Buunk et al., 2008).

Recent literature has explored the areas of divergence in mate and in-law preferences (see Apostolou, 2015b, for a review). For example, Perilloux, Fleischman, and Buss (2011) compared the students’ preferences for 13 traits in a prospective mate with their actual parents’ preferences for the same set of traits in a prospective in-law. They found that traits such as religious, good housekeeper, earning capacity, healthy, and kindness were more preferred by parents in an in-law (Perilloux et al., 2011), while physical attractiveness and exciting personality were more highly valued by offspring in a spouse. Similarly, in Chinese immigrants to North America, Hynie, Lalonde, and Lee (2006) found that traditional mate characteristics (e.g., chastity, housekeeping abilities, having strong cultural ties, and wanting children) are more preferred by the parents in an in-law than by the children in a spouse.

In another line of research, participants were asked to act as parents and express their in-law preferences and act as children and express their mate preferences. Results showed that the participants valued attractiveness and exciting personality more in a spouse than in an in-law and valued good family background and similar religious background more in an in-law than in a spouse (e.g., Apostolou, 2008a, 2008b, 2014). Because selecting an in-law of desirable backgrounds is helpful for parents to strengthen family relations and build new alliances (Apostolou, 2007a; Bejanyan et al., 2015).

Using a similar methodology, participants were given specific negative traits (i.e., unattractive and bad family background, etc.) in a potential mate, and where asked to rate whether said traits would be relatively more unacceptable to themselves or to their parents (Buunk et al., 2008; Dubbs & Buunk, 2010; Dubbs et al., 2013). Results showed that traits connoting a lack of heritable fitness (e.g., attractiveness) were considered more unacceptable to the children, while a lack of traits connoting parental investment and cooperation with in-group (e.g., good family background and similar religious background) was considered more unacceptable to their parents. Lacking traits related to romantic love (e.g., exciting personality, a sense of humor) was more acceptable to children than to parents because such traits are correlates of genetic quality and can facilitate amicable cohabitation and cooperation in love-based marriage relationships (Buunk et al., 2008).

## The Influences of Culture

Parental influences on their children’s mate selection are considerably different across cultures (Apostolou, 2010b; Bejanyan et al., 2014, 2015; Buunk et al., 2010, 2012; Dubbs et al., 2013; Hynie, Lalonde, & Lee, 2006). In individualistic cultures that advocate freedom of personal choice, mate selection is mainly based on personal preferences, stressing mutual love and attraction (Buunk et al., 2010). Individuals are encouraged to exercise personal control over their mate selection and relationship maintenance. In collectivist cultures where the well-being of the in-group is prioritized over individuals’ own needs, building new alliances is often a key objective for marriage. Parents and close kin are often involved in partner selection by directly choosing the spouse or introducing potential partners to one another (Buunk et al., 2010; MacDonald, Marshall, Gere, Shimotomai, & Lies, 2012). Children’s marriage is often utilized by their parents to forge new alliances, strengthen social standing, and continue family line (Bejanyan et al., 2014, 2015). The acceptance of a high degree of parental influence in mate selection leads the offspring to adopt and internalize parental preferences, resulting in lower levels of parents–child discrepancy.

Endorsement of traditional mate characteristics and greater parent–child convergence regarding mate preferences have indeed been observed in collectivist cultures. A cross-cultural study (Buss et al., 1990) found that the collectivists placed greater value on traditional mate characteristics such as health, desire for children, chastity, and domestic skills of the potential mates, and fewer values were given to dependability, mutual attraction, sociability, pleasing disposition, exciting personality, and appearance. Bejanyan, Marshall, and Ferenczi (2015) found that the collectivists (i.e., Indian) tended to accept greater parental influence on their mate choice, which contributed to a smaller gap between adult children’s preferences for the qualities signifying status resources in a spouse and their perception of their parents’ preferences. The collectivists also reported greater family allocentrism (Bejanyan et al., 2015), characterized as the closeness family members feel toward one another, which contributed to the smaller discrepancy between their own preferences for qualities signifying warmth trustworthiness in a spouse and their perception of their parents’ preferences.

China is a typical collectivist society (Hofstede, 1980) characterized by the ideology of filial piety that stipulates children’s obligations and duties to their parents, familial elders, and ancestors through a set of closely related behaviors, ritual practices, dispositions, and mental states (Nichols, 2013; Smith & Hung, 2012). Children often comply with parental wishes and strive to meet the expectations set by their parents, leading to the children’s mate preferences being in greater harmony with their parents’ in-law preferences than populations elsewhere in the world (Nichols, 2013). China has a long history of Confucian sex ideology in favor of male power over female. Women have been traditionally required to be submissive to men and to stay in family to support the male members of the family. Woman should be obedient to her father and older brothers before marriage, obedient to her husband after marriage, and obedient to her sons in widowhood (Chia, Allred, & Jerzak, 1997; Cook & Dong, 2011). This Confucian male-superior gender norm has been dominating China for over 2,500 years and is still influential in present society. Recent studies reveal that males’ main responsibilities are supporting the family; females’ main responsibilities are household and child-rearing tasks (Chia et al., 1997; Chen, Fiske, & Lee, 2009). In these cultural and historical settings, the mate preferences of the Chinese are expected to be different from those of the Westerners.

## The Present Study

Recent literature suggests that parental influence should be taken into consideration when applying theories of sexual selection (e.g., Buss & Schmitt, 1993; Gangestad & Simpson, 2000). Across different cultures and time, humans often have limited freedom and autonomy in mate choice (Apostolou, 2010a, b). Human mate preferences can also be substantially affected by culture. Across the 31 characteristics examined by Buss et al. (1990), culture accounted for an average of 14% of the variance. Cultural variations have also been observed in parent–child discrepancy over mating, with greater parental influence on mating in collectivist cultures than in individualistic cultures (Bejanyan et al., 2014, 2015; Buunk et al., 2010; Dubbs et al., 2013).

There is still a scarcity of literature investigating parental influence over mating in mainland China, one of the most collectivist societies (Hofstede, 1980) characterized by a high endorsement of filial piety. Unlike other collectivist values (e.g., communalism, familism), the ideology of filial piety primarily emphasizes the value of obedience of children to parents. This enables the parents to extract more resources out of their children and impose special obligations on children concerning reproductive behaviors and mate selection (Nichols, 2013). As a consequence, Chinese adult children are more likely to accept their parents’ influence or develop mate preferences similar to their parents and show more preferences for traditional marriage values than participants everywhere in the world (Buss et al., 1990; Nichols, 2013).

This is the first study designed to directly compare mate and in-law preferences in Chinese culture. Some findings are expected to be culturally universal that reflects human evolved predispositions (Apostolou, 2008a). For example, traits indicating parental investment and cooperation with the in-group should be more preferred by Chinese parents in an in-law, while traits indicating genetic quality and traits related to romantic love should be more preferred by Chinese children in a spouse (Bejanyan et al., 2014, 2015); The parents’ preferences in an in-law should also be moderated by the gender of the in-law. Most importantly, there should be cultural-specific findings that can be explained by traditional cultural values characterized by the ideology of filial piety. Specifically, there should be less parent–child divergence in mate preferences in Chinese culture than in Western cultures (Hypothesis 1). Filial piety can be used as a predictor of Chinese children’s traditional mate preferences and less parent–child divergence in mating (Hypothesis 2).

## Method

### Research Instruments

#### Factors in choosing a mate

This is a rating instrument (i.e., a normative scale) used by Buss et al. (1990). Its Chinese version was introduced by Chang, Wang, Shackelford, and Buss (2011). The participants were requested to rate each of the 18 characteristics on a 4-point scale with 3 = *indispensable* and 0 = *unimportant* according to their importance in choosing a long-term mate.

#### Preferences concerning potential mates

This is a ranking instrument (i.e., an ipsative scale) used by Buss et al. (1990) and its Chinese version was also introduced by Chang et al. (2011). The participants were requested to rank the 13 characteristics from most desirable (the rank is 1) to 13th desirable (the rank is 13) in a potential long-term mate, and each of the characteristics should be assigned a number according to their importance. Given that the ipsative scales can only capture relative rather than absolute importance of the characteristics in the scales with no variance of the total scores across participants, we primarily relied on findings in the rating instrument for comparisons. Because findings from the ranking instrument should differ predictably in magnitude, but not in direction, from findings from the rating instruments (Feingold, 1992).

#### The Filial Piety Scale (FPS)

FPS was developed by Ho (1994) to assess filial attitudes (e.g., “After the father has passed away, sons and daughters must conduct themselves according to the principles and attitudes the father followed while he was still living,” “No matter how their parents conduct themselves, sons and daughters must respect them”). All the 22 items in FPS were scored on a 7-point scale ranging from 1 (*strongly disagree*) to 7 (*strongly agree*). Cronbach’s α was .88 in this study.

### Participants and Procedure

The study was conducted under the approval of the institutional review board at Shandong Normal University. We recruited 351 undergraduates from a university in East China with students coming from all over the country. After deleting invalid cases due to incomplete responses, there were 347 undergraduates (160 sons, *M*_age_*=* 19.51, *SD*_age_*=* 1.33; 187 daughters, *M*_age_*=* 19.25, *SD*_age_*=* 0.75), 312 mothers (*M*_age_*=* 45.85, *SD*_age_*=* 2.78), and 310 fathers (*M*_age_*=* 47.06, *SD*_age_*=* 3.19) left in our data set. Among them, there were 152 son–mother dyads, 151 son–father dyads, 165 daughter–mother dyads, and 162 daughter–father dyads. Each participant received five gel pens for compensation.

After the undergraduates filled out the research instruments, they were required to send home the research instruments for their parents together with elaborate instructions. Only the rating and the ranking instruments were administrated to the parents. The male and female parents were instructed to independently indicate their preferences for an in-law in the research instruments that were required to be mailed back. The instructions for parents were transformed into the following words:

Imagine that your child who is in our university is choosing a long-term mate, you have right to express opinions on what characteristics the prospective mate should have. Please rate (or rank) these characteristics according to their importance. If your child is a daughter, you are expressing your preferences in a son-in-law; if your child is a son, you are expressing your preferences in a daughter-in-law. Your opinions are important for us.

## Results

Before data analysis, we averaged the rating/ranking scores of each child’s parents together to get a parental rating/ranking score for each trait for that child. For the single-parent child, the mother’s or the father’s scores were used as the parents’ scores. A grand mean of the items in the rating instrument but not in the ranking instrument was calculated.

### Comparing In-Law and Mate Preferences

Descriptive statistics were shown in Table 1 (the rating instrument) and Table 2 (the ranking instrument).

**Table 1.** Comparisons Between Parents and Their Actual Children (the Rating Instrument).

Characteristics	Offspring			Parents					
	Son’s *M* (*SD*)	Daughter’s *M* (*SD*)	Cohen’s *d*	Sons’ *M* (*SD*)	Daughters’ *M* (*SD*)	Cohen’s *d*	Offspring’s *M* (*SD*)	Parents’ *M* (*SD*)	Cohen’s *d*
Similar political background	.93 (.88)	.98 (.86)	.06	1.31 (.78)	1.21 (.73)	.13	.98 (.86)	1.26 (.75)	.35
Similar educational background	1.71 (.83)	1.75 (.79)	.05	2.01 (.62)	1.97 (.72)	.06	1.76 (.80)	1.99 (.67)	.31
Pleasing disposition	2.54 (.57)	2.55 (.55)	.02	2.43 (.55)	2.29 (.51)	.27	2.56 (.55)	2.36 (.54)	.37
Favorable social status	1.39 (.79)	1.86 (.67)	.65	1.80 (.67)	1.80 (.61)	.00	1.66 (.76)	1.80 (.64)	.20
Dependable character	2.78 (.43)	2.87 (.36)	.23	2.72 (.40)	2.68 (.47)	.09	2.82 (.40)	2.70 (.44)	.29
Mutual attraction–love	2.79 (.46)	2.74 (.50)	.10	2.71 (.40)	2.65 (.45)	.14	2.77 (.47)	2.68 (.43)	.20
Desire for home and children	2.23 (.88)	2.42 (.79)	.23	2.57 (.50)	2.55 (.48)	.04	2.35 (.81)	2.56 (.49)	.31
Good looks	1.63 (.68)	1.37 (.55)	.43	1.50 (.59)	1.28 (.56)	.38	1.48 (.63)	1.39 (.58)	.15
Good cook and housekeeper	1.87 (.78)	1.74 (.69)	.18	1.98 (.63)	1.59 (.59)	.64	1.81 (.74)	1.78 (.64)	.04
Ambition and industriousness	2.16 (.66)	2.66 (.51)	.86	2.29 (.55)	2.41 (.49)	.23	2.43 (.63)	2.35 (.52)	.14
Good health	2.47 (.59)	2.72 (.49)	.47	2.65 (.46)	2.69 (.44)	.09	2.61 (.55)	2.67 (.45)	.12
Good financial prospect	1.55 (.75)	2.18 (.59)	.95	1.78 (.65)	1.95 (.60)	.27	1.91 (.73)	1.87 (.63)	.06
Refinement, neatness	2.29 (.69)	2.37 (.60)	.12	2.33 (.53)	2.10 (.55)	.43	2.34 (.64)	2.21 (.55)	.22
Chastity	2.31 (.77)	2.34 (.80)	.04	2.52 (.57)	2.34 (.56)	.32	2.34 (.77)	2.42 (.57)	.12
Sociability	1.76 (.72)	2.13 (.67)	.54	1.86 (.64)	2.02 (.52)	.28	1.96 (.71)	1.94 (.59)	.03
Education and intelligence	2.15 (.62)	2.39 (.54)	.42	2.31 (.52)	2.29 (.47)	.04	2.29 (.58)	2.30 (.49)	.02
Emotional stability and maturity	1.84 (.84)	2.60 (2.32)	.42	2.23 (.53)	2.26 (.45)	.06	2.25 (1.91)	2.25 (.49)	.00
Similar religious background	.71 (.92)	.87 (.95)	.17	1.06 (.81)	1.03 (.82)	.04	.83 (.95)	1.05 (.82)	.25
Grand mean	1.95 (.33)	2.14 (.35)	.56	2.11 (.31)	2.06 (.27)	.17	2.06 (.35)	2.09 (.29)	.09

*Note. SD* = standard deviation.

**Table 2.** Comparisons Between Parents and Their Actual Children (the Ranking Instrument).

Characteristics	Offspring			Parents					
	Son’s *M* (*SD*)	Daughter’s *M* (*SD*)	Cohen’s *d*	Sons’ *M* (*SD*)	Daughters’ *M* (*SD*)	Cohen’s *d*	Offspring’s *M* (*SD*)	Parents’ *M* (*SD*)	Cohen’s *d*
Exciting personality	3.91 (2.80)	3.57 (2.88)	.12	5.35 (2.47)	5.68 (2.30)	.14	3.72 (2.84)	5.53 (2.38)	.69
Religious	11.73 (2.47)	11.86 (2.36)	.05	11.20 (2.53)	10.64 (2.68)	.22	11.80 (2.41)	10.90 (2.62)	.36
College graduate	9.54 (2.63)	8.76 (2.73)	.29	8.68 (2.18)	7.58 (2.37)	.48	9.12 (2.71)	8.09 (2.35)	.41
Easygoing	4.79 (2.81)	4.79 (2.77)	.00	5.58 (2.20)	5.93 (2.22)	.16	4.79 (2.78)	5.77 (2.21)	.39
Healthy	4.16 (2.70)	3.84 (2.57)	.12	2.91 (2.19)	3.20 (2.48)	.12	3.99 (2.63)	3.06 (2.35)	.37
Creative and artistic	9.25 (2.64)	9.20 (2.74)	.02	9.70 (2.24)	9.11 (2.19)	.27	9.22 (2.69)	9.38 (2.23)	.06
Wants children	7.90 (2.91)	9.08 (2.75)	.42	6.25 (2.31)	7.45 (2.39)	.51	8.53 (2.89)	6.90 (2.42)	.61
Good housekeeper	5.65 (2.68)	6.26 (2.85)	.22	5.35 (2.26)	6.25 (2.23)	.40	5.98 (2.79)	5.84 (2.29)	.05
Good heredity	7.26 (2.89)	6.45 (2.90)	.28	6.69 (2.32)	6.72 (2.34)	.01	6.82 (2.92)	6.71 (2.33)	.04
Physically attractive	6.91 (3.23)	8.88 (2.86)	.65	8.40 (2.41)	8.84 (2.22)	.19	7.97 (3.18)	8.64 (2.32)	.24
Kind and understanding	3.86 (2.78)	4.80 (2.80)	.34	5.06 (2.45)	5.74 (2.17)	.30	4.36 (2.83)	5.42 (2.33)	.41
Intelligent	6.05 (2.49)	5.72 (2.58)	.13	6.21 (2.35)	5.96 (2.28)	.11	5.87 (2.54)	6.08 (2.31)	.09
Good earning capacity	10.01 (2.50)	7.78 (2.98)	.81	9.61 (2.48)	7.89 (2.40)	.71	8.81 (2.98)	8.69 (2.58)	.04

*Note*. SD = standard deviation.

To analyze parent–child divergence in the 31 characteristics, General Linear Models (GLMs) were conducted with one within-subject factor (child vs. parent) and one between-subject factor (male vs. female).

Firstly, we analyzed the main effects of generation. For the rating instrument, *similar political background*, *F*(1, 311) = 23.68, *p* < .001, $\eta_{\text{p}}^{2}$ = .071, *similar educational background*, *F*(1, 311) = 21.98, *p* < .001, $\eta_{\text{p}}^{2}$ = .066, *similar religious background*, *F*(1, 311) = 13.46, *p* < .001, $\eta_{\text{p}}^{2}$ = .042, *favorable social status*, *F*(1, 311) = 10.52, *p* < .01, $\eta_{\text{p}}^{2}$ = .033, *desire for home and children*, *F*(1, 311) = 18.18, *p* < .01, $\eta_{\text{p}}^{2}$ = .055, and *good health*, *F*(1, 311) = 4.11, *p* < .05, $\eta_{\text{p}}^{2}$ = .013, were more highly valued by parents than by children. *Pleasing disposition*, *F*(1, 311) = 21.65, *p* < .001, $\eta_{\text{p}}^{2}$ = .065, *dependable character*, *F*(1, 311) = 16.49, *p* < .001, $\eta_{\text{p}}^{2}$ = .050, *mutual attraction-love*, *F*(1, 311) = 8.70, *p* < .01, $\eta_{\text{p}}^{2}$ = .027, *good looks*, *F*(1, 311) = 4.89, *p* < .05, $\eta_{\text{p}}^{2}$ = .016, and *refinement*, *neatness*, *F*(1, 311) = 9.50, *p* < .01, $\eta_{\text{p}}^{2}$ = .030, were more highly valued by children than by parents.

For the ranking instrument, *religious*, *F*(1, 345) = 29.77, *p* < .001, $\eta_{\text{p}}^{2}$ = .079, *college graduate*, *F*(1, 345) = 37.54, *p* < .001, $\eta_{\text{p}}^{2}$ = .098, *healthy*, *F*(1, 345) = 31.43, *p* < .001, $\eta_{\text{p}}^{2}$ = .084, and *wants children*, *F*(1, 345) = 85.96, *p* < .001, $\eta_{\text{p}}^{2}$ = .199, were more highly valued by parents than by children. *Exciting personality*, *F*(1, 345) = 92.49, *p* < .001, $\eta_{\text{p}}^{2}$ = .211, *easygoing*, *F*(1, 345) = 27.64, *p* <.001, $\eta_{\text{p}}^{2}$ = .074, *physically attractive*, *F*(1, 345) = 17.74, *p* <.001, $\eta_{\text{p}}^{2}$ = .049, and *kind and understanding*, *F*(1, 345) = 39.49, *p* <.001, $\eta_{\text{p}}^{2}$ = .103, were more highly valued by children than by parents.

These results indicated that the parents showed greater preferences for traditional mate characteristics than their children, while the children showed greater preferences for traits indicating genetic quality and characteristics related to romantic marriage relationships.

Then, we analyzed the main effects of gender. For the rating instrument, *favorable social status*, *F*(1, 311) = 12.47, *p* < .001, $\eta_{\text{p}}^{2}$ = .039, *ambition and industriousness*, *F*(1, 311) = 41.09, *p* < .001, $\eta_{\text{p}}^{2}$ = .117, *good health*, *F*(1, 311) = 8.68, *p* < .01, $\eta_{\text{p}}^{2}$ = .027, *good financial prospect*, *F*(1, 311) = 47.52, *p* < .001, $\eta_{\text{p}}^{2}$ = .133, *sociability*, *F*(1, 311) = 25.68, *p* < .001, $\eta_{\text{p}}^{2}$ = .076, *education and intelligence*, *F*(1, 311) = 5.61, *p* < .05, $\eta_{\text{p}}^{2}$ = .018, and *emotional stability and maturity*, *F*(1, 311) = 13.22, *p* < .001, $\eta_{\text{p}}^{2}$ = .039, were more preferred by the female participants (daughters and their parents) than by the male participants (sons and their parents). *Good looks*, *F*(1, 311) = 23.08, *p* < .001, $\eta_{\text{p}}^{2}$ = .069, and *good cook and housekeeper*, *F*(1, 311) = 19.87, *p* < .001, $\eta_{\text{p}}^{2}$ = .060, were more preferred by the male participants than by the female participants.

For the ranking instrument, *college graduate*, *F*(1, 345) = 19.88, *p* < .001, $\eta_{\text{p}}^{2}$ = .054, and *good earning capacity*, *F*(1, 345) = 80.06, *p* < .001, $\eta_{\text{p}}^{2}$ = .188, were more preferred by the female participants than by the male participants. *Wants children*, *F*(1, 345) = 29.81, *p* < .001, $\eta_{\text{p}}^{2}$ = .080, *good housekeeper*, *F*(1, 345) = 13.11, *p* < .001, $\eta_{\text{p}}^{2}$ = .037, *physically attractive*, *F*(1, 345) = 26.49, *p* < .001, $\eta_{\text{p}}^{2}$ = .071, and *kind and understanding*, *F*(1, 345) = 13.88, *p* < .001, $\eta_{\text{p}}^{2}$ = .039, were more preferred by the male participants than by the female participants.

Lastly, we analyzed the interaction between generation and gender and simple main effects following a significant interaction. For the rating instrument, the interaction effect between generation and gender on *pleasing disposition*, *F*(1, 311) = 4.19, *p* < .05, $\eta_{\text{p}}^{2}$ = .013, *favorable social status*, *F*(1, 311) = 20.48, *p* < .001, $\eta_{\text{p}}^{2}$ = .062, *dependable character*, *F*(1, 311) = 4.98, *p* < .05, $\eta_{\text{p}}^{2}$ = .016, *good cook and housekeeper*, *F*(1, 311) = 6.53, *p* <.05, $\eta_{\text{p}}^{2}$ = .021, *ambition and industriousness*, *F*(1, 311) = 26.39, *p* < .001, $\eta_{\text{p}}^{2}$ = .078, *good health*, *F*(1, 311) = 7.16, *p* < .01, $\eta_{\text{p}}^{2}$ = .023, *good financial prospect*, *F*(1, 311) = 22.42, *p* < .001, $\eta_{\text{p}}^{2}$ = .067, *refinement*, *neatness*, *F*(1, 311) = 12.80, *p* < .001, $\eta_{\text{p}}^{2}$ = .040, *chastity*, *F*(1, 311) = 4.34, *p* < .05, $\eta_{\text{p}}^{2}$ = .014, *sociability*, *F*(1, 311) = 6.02, *p* < .05, $\eta_{\text{p}}^{2}$ = .019, *education and intelligence*, *F*(1, 311) = 9.02, *p* < .01, $\eta_{\text{p}}^{2}$ = .028, and *emotional stability and maturity*, *F*(1, 311) = 12.48, *p* < .001, $\eta_{\text{p}}^{2}$ = .041, were significant.

Simple main effect analysis showed that *pleasing disposition*, *F*(1, 311) = 5.97, *p* < .05, $\eta_{\text{p}}^{2}$ = .019, *good cook and housekeeper*, *F*(1, 311) = 31.71, *p* < .001, $\eta_{\text{p}}^{2}$ = .093, *refinement*, *neatness*, *F*(1, 311) = 13.55, *p* < .001, $\eta_{\text{p}}^{2}$ = .043, and *chastity*, *F*(1, 311) = 7.73, *p* < .01, $\eta_{\text{p}}^{2}$ = .024, were more highly valued by the sons’ parents than by the daughters’ parents in an in-law while they were almost equally valued by sons and daughters in a spouse. *Favorable social status*, *F*(1, 311) = 27.54, *p* < .001, $\eta_{\text{p}}^{2}$ = .081, *dependable character*, *F*(1, 311) = 3.93, *p* < .05, $\eta_{\text{p}}^{2}$ = .012, *ambition and industriousness*, *F*(1, 311) = 60.86, *p* < .001, $\eta_{\text{p}}^{2}$ = .164, *good health*, *F*(1, 311) = 13.24, *p* < .001, $\eta_{\text{p}}^{2}$ = .041, *education and intelligence*, *F*(1, 311) = 12.40, *p* < .001, $\eta_{\text{p}}^{2}$ = .038, and *emotional stability and maturity*, *F*(1, 311) = 13.73, *p* < .001, $\eta_{\text{p}}^{2}$ = .042, were more highly valued by daughters than by sons while the difference between daughter’s parents and son’s parents was nonsignificant.

For the ranking instrument, the interaction effect between generation and gender on *religious*, *F*(1, 345) = 4.56, *p* < .05, $\eta_{\text{p}}^{2}$ = .013, *good heredity*, *F*(1, 345) = 4.86, *p* < .05, $\eta_{\text{p}}^{2}$ = .014, and *physically attractive*, *F*(1, 345) = 19.89, *p* < .001, $\eta_{\text{p}}^{2}$ = .055, were significant. Simple main effect analysis showed that *religious* were more highly valued by the daughters’ parents than by the sons’ parents in an in-law, *F*(1, 345) = 3.91, *p* < .05, $\eta_{\text{p}}^{2}$ = .011, while it was almost equally valued by sons and daughters in a spouse. *Good heredity* was more highly valued by sons in a spouse than by their parents in an in-law, *F*(1, 345) = 4.14, *p* < .05, $\eta_{\text{p}}^{2}$ = .012], but the difference between daughters and their parents was not significant. *Physically attractive* was much more highly valued by sons than by daughters [*F*(1, 345) = 36.42, *p* < .001, $\eta_{\text{p}}^{2}$ = .095, but the difference between the sons’ and daughters’ parents was not significant.

A GLM analysis of the grand mean of the rating instrument revealed a significant gender effect, *F*(1, 311) = 5.47, *p* < .05, $\eta_{\text{p}}^{2}$ = .017, and a significant Generation × Gender interaction, *F*(1, 311) = 33.78, *p* < .001, $\eta_{\text{p}}^{2}$ = .099. Simple main effects analysis indicated that daughters are choosier in mate selection compared with sons, *F*(1, 311) = 24.14, *p* < .001, $\eta_{\text{p}}^{2}$ = .071. But the daughters’ parents were not more demanding than the sons’ parents on an in-law. This should be treated as another heritage of traditional Chinese cultural values.

### Filial Piety as a Predictor of Preference for Traditional Traits (PTTs) and Less Parent–Child Divergence

Correlations between filial piety and individual items are shown in Table 3 (the rating instrument) and Table 4 (the ranking instrument).

**Table 3.** Correlations Between Filial Piety and Parent–Offspring Divergence (Rating Instrument).

Characteristics	Offspring	Father–Offspring Difference	Mother–Offspring Difference	Parent–Offspring Difference
Similar political background	.12*	−.05	−.13*	−.11
Similar educational background	−.01	−.03	−.07	−.04
Pleasing disposition	.06	−.04	.01	.00
Favorable social status	−.03	−.02	.01	.00
Dependable character	.08	−.02	.02	.00
Mutual attraction–love	.09	−.09	.02	−.04
Desire for home and children	.24**	−.17**	.02	−.18**
Good looks	−.05	.00	−.12*	−.06
Good cook and housekeeper	.15**	−.05	.02	.03
Ambition and industriousness	.01	−.01	−.06	.03
Good health	−.04	.06	−.02	.03
Good financial prospect	.02	.04	−.03	.01
Refinement, neatness	−.01	−.01	−.07	−.02
Chastity	.27**	−.16**	−.17**	−.18**
Sociability	.12*	−.15**	−.06	−.13*
Education and intelligence	−.11*	.02	.07	.02
Emotional stability and maturity	.05	.01	.01	.01
Similar religious background	.03	.06	−.07	−.02

*Note*. *p < .05. **p < .01

**Table 4.** Correlations Between Filial Piety and Parent–Offspring Divergence (Ranking Instrument).

Characteristics	Offspring	Father–Offspring Difference	Mother–Offspring Difference	Parent–Offspring Difference
Exciting personality	.17**	.03	−.02	.01
Religious	−.02	.04	−.06	.00
College graduate	.11	−.02	−.01	−.00
Easygoing	.06	−.01	−.02	−.02
Healthy	−.10	−.11	−.06	−.09
Creative and artistic	.18**	−.15^*^	−.09	−.12*
Wants children	−.23**	−.08	−.12^*^	−.13*
Good housekeeper	−.03	−.03	−.12^*^	−.04
Good heredity	.03	−.10	.04	−.02
Physically attractive	.08	.03	.00	.02
Kind and understanding	.01	.01	−.11	−.05
Intelligent	.15**	−.09	−.01	−.08
Good earning capacity	.03	−.13*	.05	−.06

*p < .05. **p < .01

We predicted that in Chinese culture, there is less parent–child divergence regarding what constitute an ideal mate for the children. Spearman’s correlation and effect sizes (Cohen, 1992) were calculated as the indicators of overall parent–child convergence in mate preference. In the rating instrument, the correlation between parents and children, between parents and sons, and between parents and daughters were .94, .96, and .93, respectively (Cohen’s *d* = .23, .32, and .25). In the ranking instrument, the correlations were .95, .94, and .94, respectively (Cohen’s *d* = .30, .37, and .32). These correlation coefficients were higher than those we calculated using the data of an American sample reported by Perilloux et al. (2011), which were .71, .76, and .70, respectively (Cohen’s *d* = .31, .54, and .27). A medium effect size (Cohen, 1992) was only observed in the American sample. These results suggested more parent–child convergence in Chinese culture.

We further hypothesized that more parent–child convergence in Chinese culture can be attributed to children’s PTTs, which may be explained by filial piety. Based on the previous literature (Buss et al., 1990; Hynie et al., 2006), we used 10 characteristics (*similar political background*, *similar educational background*, *favorable social status*, *desire for home and children*, *good cook and housekeeper*, *good health*, *good financial prospect*, *chastity*, *sociability*, and *similar religious background*) to measure the PTTs. These items were averaged to create a PTT Scale, with internal consistency (Cronbach’s α) acceptable both for parents (α = .70) and for children (α = .70). Results showed that the children’s filial piety scores was related to their PTT scores, *r* = .18, *p* < .01 (two-tailed), but was not related to their parents’ PTT scores, *r* = −.04, *p* = .49 (two-tailed). Filial piety also correlated negatively with the absolute parent–child PTT score difference, *r* = −.16, *p* < .01 (two-tailed). For items in the rating instrument, filial piety correlated positively with *similar political background*, *r* = .12, *p* < .05 (two-tailed), *desire for home and children*, *r* = .24, *p* < .001 (two-tailed), *good cook and housekeeper*, *r* = .15, *p* < .01 (two-tailed), *chastity*, *r* = .27, *p* < .001 (two-tailed), and *sociability*, *r* = .12, *p* < .05 (two-tailed); and negatively with *education and intelligence*, *r* = −.11, *p* < .05 (two-tailed). Filial piety also correlated negatively with the absolute parent–child score difference in *desire for home and children*, *r* = −.18, *p* < .01 (two-tailed), *chastity*, *r* = −.18, *p* < .01 (two-tailed), and *sociability*, *r* = −.13, *p* < .05 (two-tailed).

For the ranking instrument, filial piety correlated negatively with *wants children*, *r* = −.23, *p* < .001 (two-tailed), and positively with *exciting personality*, *r* = .17, *p* < .01 (two-tailed), *creative and artistic*, *r* = .18, *p* < .01 (two-tailed), and *intelligence*, *r* =.15, *p* < .01 (two-tailed). Filial piety also correlated negatively with the absolute parent–child score difference in *creative and artistic*, *r* = −.12, *p* < .05 (two-tailed), and *wants children*, *r* = −.13, *p* < .05 (two-tailed). But we failed to create a reliable scale measuring the PTTs.

These results indicated that the children who endorsed the ideology of filial piety were more likely to accept traditional marriage values and develop mate preferences similar to their parents and were less likely to value characteristics indicating genetic quality and traits related to romantic love.

## Discussion

In human history, evolutionary pressures have resulted in in-law and mate preferences to diverge (Gangestad, Haselton, & Buss, 2006). Both parents and children have evolved mating preferences which enable the two parties to select mates and in-laws to maximize the inclusive fitness of their own (Apostolou, 2007b, 2008a, 2008b; Buunk et al., 2008; Perilloux et al., 2011). Results in this study are consistent with these findings. Traits indicating parental investment and traditional values were more highly valued by the parents, while traits indicating genetic quality and romantic love were more highly valued by the children. Chinese parents preferred characteristics such as the *similar religious background* in an in-law more so than the children in a spouse. Chinese children preferred characteristics such as *dependable character* and *good looks* in a spouse more so than the parents in an in-law. *Good financial prospect* and *sociability* were more preferred in a son-in-law than in a daughter-in-law by the parents. Traits connoting genetic quality and reproductive fitness (e.g., *physical attractiveness*) and traits related to family care (e.g., *good cook and housekeeper*) were more preferred by the parents for a daughter-in-law than a son-in-law. These results suggested that the nature of parent–child divergence over mating may be a cultural universal.

### Less Divergence Between In-law and Mate Preferences and the Role of Traditional Cultural Values

However, this study also has revealed some culture-specific findings that should be interpreted by Chinese traditional cultural values. This study found more parent–child convergences in mate preferences compared with Western samples (Perilloux et al., 2011). This is in line with previous findings that East Asians tend to accept more parental influence on mate choice than Europeans and European Americans (Buunk et al., 2010, 2012; Nichols, 2013). Endorsement of filial piety can lead Chinese children to accept traditional marriage values, resulting in less parent–child divergence in mating. This finding is further supported by literature showing that Taiwanese adolescents with stronger filial beliefs were found to be less likely to experience parent–child conflict (Yeh & Bedford, 2004). Therefore, both Hypotheses 1 and 2 were supported.

In China, the disagreement in in-law and mate preferences can be handled effectively in a cultural-specific way. That is, parent–offspring conflict over mating that arises from diverging genetic interests can be handled in a way that it does not escalate. Through the internalization of filial obligations, children are more willing to accept the traditional marriage values (continuing family line, enlarging family resources, improving social status, and bringing honor to ancestors) and are less committed to love-based marriages. The desire for chastity in a potential mate is a good testimony to show the internalization of parental preferences in Chinese children’s mating behaviors. Buss et al. (1990) observed stronger desirability for chastity in a potential mate in both Chinese males and females, indicating stronger traditional mate preferences than the participants everywhere in the world.

### The Daughters’ Parents Were Not More Demanding Than the Sons’ Parents

Parents are supposed to be more demanding in selecting a son-in-law than a daughter-in-law (Buunk et al., 2008). For example, Apostolou (2010a) investigated 67 preindustrial societies and found that parents valued wealth, working ability, social status, and good economic prospects more in a son-in-law than in a daughter-in-law. Only chastity was valued by the parents more in a daughter-in-law than in a son-in-law.

But in this study, the daughters’ parents were even less demanding than the son’s parents (though the difference was not significant). There are evolutionary explanations for this phenomenon. Parents are expected to be more vigilant particularly about a daughter’s early sexual activity because it is more likely to drain their own resources if it leads to a child out of “wedlock” (Perilloux et al., 2008). But this does not necessarily result in more choosiness in selecting a long-term mate for a daughter. On the other hand, the sons’ parents could also be stricter in in-law selection. If a daughter-in-law is unfaithful or does not take good care of her offspring, the parents may run the risk of losing all investments in their son (Buss & Schmitt, 1993).

But Chinese culture should also be taken into account when understanding this phenomenon. After a female is married, she becomes a member of her husband’s family unit (this is seldom the case for a male) and takes the responsibility to bear children to continue the family line of her husband’s paternal ancestors (Nichols, 2013; Smith & Hung, 2012). If a daughter-in-law is well educated and comes from good family background, she is expected to be capable of rearing high-quality children. Moreover, in traditional China, wealth was inherited through the male line from father to son (Nichols, 2013; Smith & Hung, 2012). A virtuous (e.g., chaste, filial, and industrious) daughter-in-law played a key role in enhancing cooperation with in-group, maintaining and enlarging family property, and raising family standing. Additionally, when the parents get older and gradually lose control over family resources, they mainly depend on daughter-in-laws for elderly care (Cook & Dong, 2011).

### Other Culture-specific Findings

This study has revealed some interesting findings that should be interpreted by Chinese traditional values and gender role ideology. First, *desire for home and children* and *healthy* were more preferred by the parents in an in-law than by the children in a spouse, while in Western cultures (e.g., Perilloux et al., 2011) this phenomenon has not been observed. These results again revealed that Chinese parents prefer in-laws with traditional traits in order to continue ancestral line (Hynie et al., 2006). In China, the piety of a son is proportional to the number of and quality of children, and bearing a male heir was the son’s highest duty. The *desire for children* is also preferred by the daughters’ parents in a son-in-law because their daughters’ marriages might be stabilized by the birth of babies. Second, *kind and understanding* was more preferred by parents than by children and was more preferred by daughters than by sons in Western samples (Perilloux et al., 2011). As a kind mate is more willing to dedicate time and energy in childcare. But in this study, *kind and understanding* was less preferred by parents than by children and was less preferred by female than by male participants. This suggests that kindness was less valued by parents (especially the daughters’) than by children. For the parents (especially the daughters’), generosity of an in-law may result in resources being allocated to genetically unrelated ones (Guo, Feng, & Wang, 2017; Oda, Shibata, Kiyonari, Takeda, & Matsumoto-Oda, 2013). But for the children, kindness is important for love-based marriage because it can promote a harmonious partnership and amicable cohabitation (Apostolou, 2008b). Third, Western parents prefer working ability and social status more in a son-in-law than in a daughter-in-law (Apostolou, 2007a, 2008a, 2008b), but in this study these differences have not been observed, given that the in-laws’ *social status* and *ambition and industries* were highly valued both by the sons’ and daughter’s parents. This can also be interpreted as the influence of traditional culture values and gender role socialization in Chinese society. An industrious, high social status daughter-in-law is capable of rearing high-quality children and taking care of family elders, these characteristics are especially valuable for the son’s parents (Nichols, 2013; Smith & Hung, 2012).

Findings in this study add to the literature illustrating how cultural values have shaped human mating behaviors (Buunk et al., 2010). Humans have evolved psychological adaptations that are specifically designed to receive and process variable social and cultural input. Thus, they are exceptionally responsive to the cultural environments they are exposed to (Gangestad et al., 2006). Taking into account parental influences, social and ecological conditions may enable us to more accurately understand the evolution of human mating behavior (Apostolou, 2007b).

Some findings in this study may not be specific to Chinese ethnic groups. Literature shows that filial piety, familism, and communalism share a common thread in stressing the importance of social ties over individual interests and valuing the needs of the in-group over the needs of individual person (Schwartz et al., 2010). We infer that people from other collectivist cultures may also be more likely to accept traditional mate values and have mate preferences more similar to their parents’ in-law preferences. This may be particularly the case in places where people live near their parents and extended families and depend on kin. Even in Western samples, the participants who are more concerned about their parents’ opinions are more likely to shift their mate preferences to conform to those of their parents (Dubbs et al., 2012). That is, the individuals’ sensitivity toward parents is positively linked with their preferences for parental investment and cooperation in a potential mate.

This work is not without limitations. One being that it examined in-law preferences using instruments containing a greater number of traditional/parental investment traits, compared to traits indicative of genetic quality. Future studies should include more traits that in theory children would prefer in a mate. Furthermore, this study found that Chinese children are socialized to accept traditional marriage values and develop mate preferences similar to their parents. This proposition was supported by the positive correlation between filial piety and traditional mate preference. We further proposed that filial piety should be negatively correlated with the preferences for characteristics indicating genetic quality and traits related to romantic love. However, using the items in the research instruments in this study, we failed to construct a reliable scale measuring the preference for genetic quality and the preference for traits related to romantic love. A future study is encouraged to address this issue using items developed in recent literature (e.g., Apostolou, 2015b; Chang, Lu, & Zhu, 2017).

Another limitation is that this study was conducted only in Chinese culture setting, leaving in-law and mate preferences not being directly compared in other collectivist cultures, such as Africa, Arab countries, India, and Spanish-speaking countries (Schwartz et al., 2010). Future work needs to replicate these findings in different samples and in different cultural settings. Furthermore, in this study, filial piety only showed a rather limited power in predicting parent–child convergence in mate preferences. One reason may be that the FPS (Ho, 1994) used in this study only measures filial belief, which might rarely be reflected in actual behaviors (Chow & Chu, 2007). Another reason is that filial piety may be a multidimensional construct (Yeh & Bedford, 2003, 2004). Future researchers are expected to explore whether multidimensional filial piety construct can better predict the parent–child agreement over mating. Additionally, findings in this study were based on self-report data, we are not sure whether filial piety can reduce parent–offspring divergence in real-life situations. Future naturalistic studies may overcome this limitation.

Previous literature also suggests that mate values of an individual who excises mate selection also influence the mate preferences of that individual and his or her parents (Apostolou, 2011; Li, Bailey, Kenrick, & Linsenmeier, 2002). Future studies should take into account mate values of an individual (e.g., physical attractiveness, height, health, income, social status, education, and personality) and those of his or her parents when examining parent–child convergence and divergence over mating (Chang et al., 2017). Social–economic status and size of a family also influence the parents’ ability to invest into their child and grandchildren (Lawson & Mace, 2010). These factors are recommended to be included in future studies.

This is the first study that directly compared in-law and mate preferences in Chinese culture, illustrating how parent–child disagreement over mating could be handled in a culturally specific way. In China and probably other collectivist societies, children are socialized to adopt and internalize parental preferences, resulting in low level of divergence in mate and in-law preferences. More specifically, traditional culture values (e.g., filial piety) can handle the parent–child disagreement over mating that arises from diverging genetic interests in a way that it does not escalate.
